# Increased MicroRNA Levels in Women With Polycystic Ovarian Syndrome but Without Insulin Resistance: A Pilot Prospective Study

**DOI:** 10.3389/fendo.2020.571357

**Published:** 2020-09-30

**Authors:** Alexandra E. Butler, Vimal Ramachandran, Thomas Keith Cunningham, Rhiannon David, Nigel J. Gooderham, Manasi Benurwar, Soha R. Dargham, Shahina Hayat, Thozhukat Sathyapalan, S Hani Najafi-Shoushtari, Stephen L. Atkin

**Affiliations:** ^1^Diabetes Research Center (DRC), Qatar Biomedical Research Institute (QBRI), Hamad Bin Khalifa University (HBKU), Qatar Foundation (QF), Doha, Qatar; ^2^Division of Research, Weill Cornell Medicine-Qatar, Qatar Foundation, Education City, Doha, Qatar; ^3^Academic Diabetes, Endocrinology and Metabolism, Hull York Medical School, University of Hull, Heslington, United Kingdom; ^4^Department of Surgery & Cancer, Faculty of Medicine, Imperial College, London, United Kingdom; ^5^Department of Cell and Developmental Biology, Weill Cornell Medicine, New York, NY, United States; ^6^Postgraduate Studies and Research, Royal College of Surgeons Ireland, Al Muharraq, Bahrain

**Keywords:** microRNA, polycystic ovary syndrome, non-obese, insulin sensitivity, anti-Mullerian hormone, female

## Abstract

**Background:**

Small noncoding microRNA (miRNA) have regulatory functions in polycystic ovary syndrome (PCOS) that differ to those in women without PCOS. However, little is known about miRNA expression in women with PCOS who are not insulin resistant (IR).

**Methods:**

Circulating miRNAs were measured using quantitative polymerase chain reaction (qPCR) in 24 non-obese BMI and age matched women with PCOS and 24 control women. A miRNA data set was used to determine miRNA levels.

**Results:**

Women with PCOS showed a higher free androgen index (FAI) and anti-mullerian hormone (AMH) but IR did not differ. Four miRNAs (miR-1260a, miR-18b-5p, miR-424-5p, and miR let-7b-3p) differed between control and PCOS women that passed the false discovery rate (FDR) out of a total of 177 circulating miRNAs that were detected. MiRNA let-7b-3p correlated with AMH in PCOS (p < 0.05). When the groups were combined, miR-1260a correlated with FAI and let-7b-3p correlated with body mass index (BMI) (p < 0.05). There was no correlation to androgen levels. Ingenuity pathway analysis showed that nine of the top 10 miRNAs reported were associated with inflammatory pathways.

**Conclusion:**

When IR did not differ between PCOS and control women, only four miRNA differed significantly suggesting that IR may be a driver for many of the miRNA changes reported. Let-7b-3p was related to AMH in PCOS, and to BMI as a group, whilst miR-1260a correlated with FAI. Androgen levels, however, had no effect upon circulating miRNA profiles. The expressed miRNAs were associated with the inflammatory pathway involving TNF and IL6.

## Introduction

In females of reproductive age, polycystic ovarian syndrome (PCOS) is the leading disorder of the endocrine system, affecting 9–21% of females and PCOS is a major cause of anovulatory infertility (1). Characteristic findings in PCOS include obesity, type 2 diabetes, hyperandrogenism, insulin resistance (IR), and hypercholesterolemia (2). Genetic factors, such as mutations, and changes in epigenetics and/or expression of non-coding RNAs, are thought to play a significant role in the etiology of PCOS (3–5).

MiRNAs are short (transcripts are ~22 nucleotides long) endogenous non-coding RNAs that bind (6) *via* their first eight bases to messenger RNA (mRNA) transcripts, resulting in post-transcriptional regulation of gene expression through mRNA degradation and translational repression (7–10). MiRNAs attenuate the expression of a multitude of target mRNAs regulating biological and cellular pathways. It is, therefore, not surprising that miRNAs play a role in obesity and related conditions such as insulin resistance, glucose intolerance, type 2 diabetes and dyslipidemias (11–15).

MiRNA expression in the serum of PCOS women is therefore likely to be complex, and studies in the literature appear discordant (16). It is reported that miR-21, miR-27b, miR-103, and miR-155 expression is altered in both obesity and in PCOS, and therefore indirectly in IR (17). MiR-93 has also been implicated in downregulating the expression of *SLC2A4*, an insulin-sensitive glucose transporter, in adipose tissue (18) where it is overexpressed in women with PCOS with insulin resistance (19), though how this relates to the circulating miRNA is unclear. Others have reported several tissue related miRNA to be associated with IR (6), examples being miR-135 in endothelial cells (20) and let7 in muscle cells (21).

It is therefore clear that miRNAs are related to insulin resistance, but no study has been undertaken to date to determine if miRNAs differ in a PCOS population that was not insulin resistant compared to a control population.

## Materials and Methods

### Study Design

This was a prospective cohort study and was performed from January 2014 to January 2016 within the Hull Hospitals following approval by the Yorkshire and The Humber NRES ethical committee, UK, and all women gave their written informed consent. All women attending the IVF clinic for treatment of their subfertility by means of assisted conception were approached, of whom 60 subjects (30 PCOS and 30 controls) agreed to participate and were concurrently recruited. Forty-eight women, 24 PCOS subjects and 24 normal controls, aged between 20–44 years who fulfilled the criteria of not having insulin resistance were included in the study (HOMA-IR less than 2). For the diagnosis of PCOS, two of three diagnostic criteria of the Rotterdam consensus were used; these criteria are (1) clinical and biochemical hyperandrogenemia, requiring a Ferriman-Gallwey score of >8 and a free androgen index of >4 respectively, (2) oligomenorrhea or amenorrhea and (3) polycystic ovaries seen on transvaginal ultrasound (22). Study participants had no other condition or illness and all women were on folic acid 400 µg daily, but no other medication. Testing was undertaken to ensure that no patient had any of the following endocrine conditions: non-classical 21-hydroxylase deficiency, hyperprolactinaemia, Cushing’s disease or an androgen-secreting tumour. Control women were age and body mass index (BMI) matched to the PCOS patients. Demographic data for both control and PCOS women is shown in **Table 1**.

**Table 1:** Characteristics of control and PCOS subjects showing that whilst matched for age and body mass index, PCOS subjects had higher androgen (free androgen index) and anti-Mullerian hormone (AMH) levels.

	Control women (n = 24)	PCOS women (n = 24)	P-value
	Mean ± SD	Mean ± SD	
Age (years)^a^	32.5 ± 4.1	31.0 ± 6.4	0.21
BMI^a^	24.8 ± 1.1	25.9 ± 1.8	0.26
Glucose (mmo/l)^b^	4.8 ± 0.4	4.7 ± 0.4	0.07
Insulin (mIU/ml)^b^	7.7 ± 4.2	8.3 ± 4.7	0.60
HOMA IR^b^	1.7 ± 1.0	1.8 ± 1.0	0.83
FAI^b^	1.3 ± 0.5	4.1 ± 2.9	0.001
AMH (ng/ml)^b^	24 ± 13	57 ± 14	0.001

BMI, body mass index; FAI, free androgen index (= testosterone/sex hormone binding globulin x 100); HOMA-IR, homeostatic model assessment-insulin resistance). AMH, antimullarian hormone. Anti-Müllerian hormone was measured using a Beckman Coulter Access automated immunoassay.

^a^T-test used; ^b^Mann-Whitney test used.

The primary endpoint was the expression of miRNA between non-insulin resistant PCOS and age and BMI matched control subjects.

### Sample Collection

Following an overnight fast, blood samples were taken in the follicular phase of the cycle. Serum was collected and stored frozen at -80°C for batch analysis. Serum testosterone was measured by isotope dilution liquid chromatography- tandem mass spectrometry (Waters Corporation, Manchester, UK). Sex hormone binding globulin (SHBG) was determined using an immunometric assay (following the manufacturer-recommended protocol), the fluorescence being detected using a DPC Immulite 2000 analyzer. The free androgen index (FAI) was calculated from the formula: total testosterone x100/SHBG. Serum insulin was determined by competitive chemiluminescent immunoassay using a DPC Immulite 2000 analyzer (Euro/DPC, Llanberis, UK) following the manufacturer-recommended protocol with no stated cross-reactivity with proinsulin; analytical sensitivity was 2 µU/ml and the coefficient of variation (CV) was 6%. Plasma glucose was measured using the manufacturer-recommended protocol on a Synchron LX 20 analyzer (Beckman-Coulter) with a CV of 1.2% at the mean glucose concentration of 5.3mmol/liter. Insulin resistance was calculated using the standard HOMA method [HOMA-IR = (insulin x glucose)/22.5]. Anti-Müllerian hormone was measured using a Beckman Coulter Access automated immunoassay; between run precision was <3% across the range measured (23).

### MiRNA Profiling and Analysis

Total RNA was isolated from 200 µl plasma of each sample using the miRCURY RNA Isolation Kit - Biofluids (Exiqon; acquired by Qiagen) following the manufacturer-recommended protocol. An on-column DNase digestion was performed for 15 min during the RNA extraction procedure based on manufacturer guidelines to remove contaminating DNA. Since precise estimation of RNA concentration from biofluids such as plasma is difficult (24), reverse transcription was performed with equal volumes of RNA from each sample (4 µl of RNA in 20 µl reverse transcription reaction volume) using Exiqon Universal cDNA Synthesis Kit II following the manufacturer’s protocol. Such a method of inputting equal volumes of total RNA into the cDNA synthesis reaction has been described earlier (25). RNA integrity and reverse transcription efficiency were assessed using representative samples that were loaded on to miRNA QC PCR Panel (Exiqon). Quality control was ensured by monitoring diagnostic assays for miRNAs that are expressed in various tissues and present on the QC panel. Additionally, the QC panel also contains assays for the synthetic RNA isolation spike-ins, UniSp2, 4 and 5 (Exiqon), that were added prior to RNA isolation and the cDNA synthesis spike-ins, UniSP6 and cel-miR-39-3p (Exiqon), added prior to reverse transcription. To perform qPCR, 2x Exilent SYBR Green master mix (Exiqon) was mixed with fifty-fold diluted cDNA and 4 μl per 2 ml ROX Reference Dye (ThermoFisher Scientific) before loading on to Exiqon Serum/Plasma Focus microRNA PCR Panel, 384 well (V4.M). The Exiqon Serum/Plasma Focus microRNA PCR Panel used for miRNA profiling in this study contains primer sets for 179 circulating miRNAs in addition to assays for synthetic spike-ins and reference miRNAs. Amplification was performed using QuantStudio 12K Flex Real-Time PCR System (ThermoFisher Scientific) followed by pre-processing of raw data and statistical analysis using GenEx qPCR analysis software, version 6 (MultiD). Pre-processing of the raw data was conducted as follows. Inter-panel differences among runs were normalized with the UniSp3 spike-in contained on the panels. False positive amplifications were eliminated by running a no-template negative control panel and then setting a ΔCt of 1 between the sample and negative control for every miRNA assayed. To identify and leave out haemolysed samples, a ΔCt >7 between hsa-miR-23a-3p and hsa-miR-451a was set as a cutoff as described (26, 27). Data was normalized against the global mean of all expressed miRNAs with a Ct less than 35. Upon completion of pre-processing, samples were divided into two groups for statistical analysis, PCOS, and controls.

### Ingenuity Pathway Analysis

Ingenuity Pathways Analysis (Qiagen, Redwood City, USA) was undertaken on the miRNA data. IPA is a web-based bioinformatics application in which the miRNA data is upload and that then generates lists of genes and allows interactive building of networks to represent biological systems. Pathways overrepresented by the FDR-significant miRNA changes at q<0.05 (28) were identified.

### Statistics

No data on PCOS without insulin resistance and miRNA expression were available to undertake a formal power analysis on changes in miRNA expression levels; therefore, this study was conducted as a pilot study. Birkett and Day (29) have suggested a minimum of 20 degrees-of-freedom to allow an estimation of variance from which a larger trial could be powered; therefore, in this study, 24 subjects in each group were recruited. Statistical analysis was calculated using SPSS (v22, Chicago, Illinois). Data presentation: for continuous data, the descriptive data is here presented as mean ± SD. Students t-test was used to compare between means; Mann Whitney tests were used for data that violated the assumptions of normality when tested using the Kolmogorov-Smirnov test. Spearman’s correlation test was used to assess associations. An unpaired two-tailed t-test with Bonferroni correction was used in order to test any paired changes in miRNA expression levels between the PCOS and control women. False discovery rates (FDR) of q<0.05 (28) were taken as significant.

## Results

Baseline data for the 24 PCOS patients and the 24 control patients are shown in **Table 1**, where it can be seen that the subjects were non-obese, matched for age and BMI, and were not insulin resistant.

**Figure 1:**
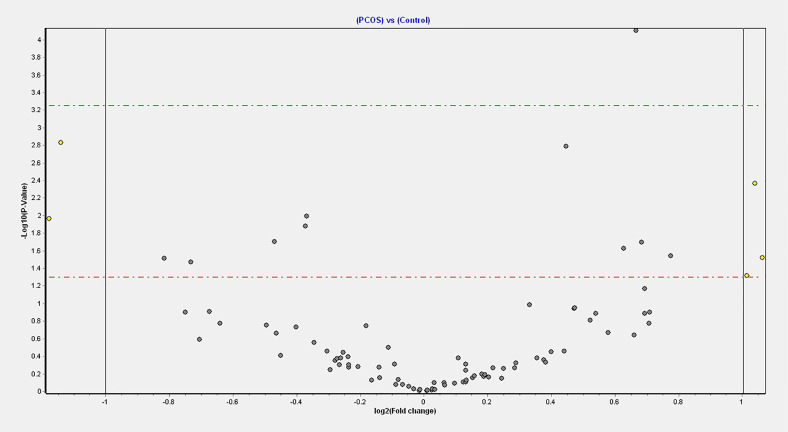
Volcano plot of fold changes in the key miRNAs that passed the FDR threshold and significance level.

Four miRNAs passed the false discovery rate (FDR) and differed between the PCOS subjects and controls, as shown in **Table 2** and **Figure 1**. A total of 177 circulating miRNAs were detected (shown in **Supplemental Table 1**); the 4 miRNA that passed FDR p<0.05 that differed between normal women and PCOS were miR-1260a (p = 0.015), miR-18b-5p (p = 0.029), miR-424-5p (p = 0.038), and let-7b-3p (p = 0.042). **Table 3** shows the correlation of the demographic features with the miRNAs. Let-7b-3p correlated with AMH in PCOS (p < 0.05), and when the groups were combined miR-1260a correlated with free androgen index (FAI) and AMH; let-7b-3p correlated with BMI (p < 0.05).

**Table 2:** Top 10 miRNA detected, four of which differed significantly between patients with and without PCOS matched for age and BMI.

PCOS vs controls	Fold change	FDR corrected P-Value
hsa-miR-1260a	1.945	0.015
hsa-miR-18b-5p	1.323	0.029
hsa-miR-424-5p	-1.406	0.038
hsa-let-7b-3p	1.485	0.042
hsa-miR-2110	-1.389	0.056
hsa-let-7c-5p	-1.376	0.075
hsa-miR-454-3p	-1.438	0.075
hsa-miR-130a-3p	1.185	0.076
hsa-miR-150-5p	-1.390	0.098
hsa-miR-133b	1.730	0.113

Positive fold change indicates that the miRNA is upregulated in PCOS women compared to controls. Negative fold change indicates that the miRNA is downregulated in PCOS women compared to controls.

**Table 3:** Spearman correlations of demographics with top four significant miRNAs for both control and PCOS patients, control and PCOS patients alone.

	Age	BMI	AMH	Fertility rate	HOMA-IR	FAI
**All patients**						
**hsa-let-7b-3p**	-0.148	0.261^*^	-0.159	0.142	-0.094	-0.090
**hsa-miR-1260a**	-0.110	-0.041	-0.349^**^	-0.143	-0.161	-0.331^*^
**hsa-miR-424-5p**	-0.220	0.143	0.100	0.049	-0.114	0.140
**hsa-miR-18b-5p**	-0.044	0.110	-0.195	0.026	-0.185	-0.093
**Control patients**						
**hsa-let-7b-3p**	-0.311	0.392^*^	0.052	0.233	-0.137	0.018
**hsa-miR-1260a**	-0.179	0.116	-0.153	0.029	-0.116	-0.203
**hsa-miR-424-5p**	-0.295	0.225	-0.048	0.095	-0.223	-0.103
**hsa-miR-18b-5p**	-0.218	0.276	-0.191	0.122	-0.225	-0.091
**PCOS patients**						
**hsa-let-7b-3p**	0.041	0.113	-0.408^*^	-0.015	-0.038	-0.063
**hsa-miR-1260a**	-0.208	-0.152	-0.212	-0.330	-0.158	-0.263
**hsa-miR-424-5p**	-0.057	-0.002	-0.044	-0.169	-0.075	0.130
**hsa-miR-18b-5p**	0.192	-0.131	-0.348	-0.191	-0.179	-0.129

BMI, body mass index; AMH, antimullerian Hormone; HOMA-IR, homeostatic model assessment-insulin resistance; FAI, free androgen index.

*P-value < 0.05; **P-value < 0.001.

**Figure 2:**
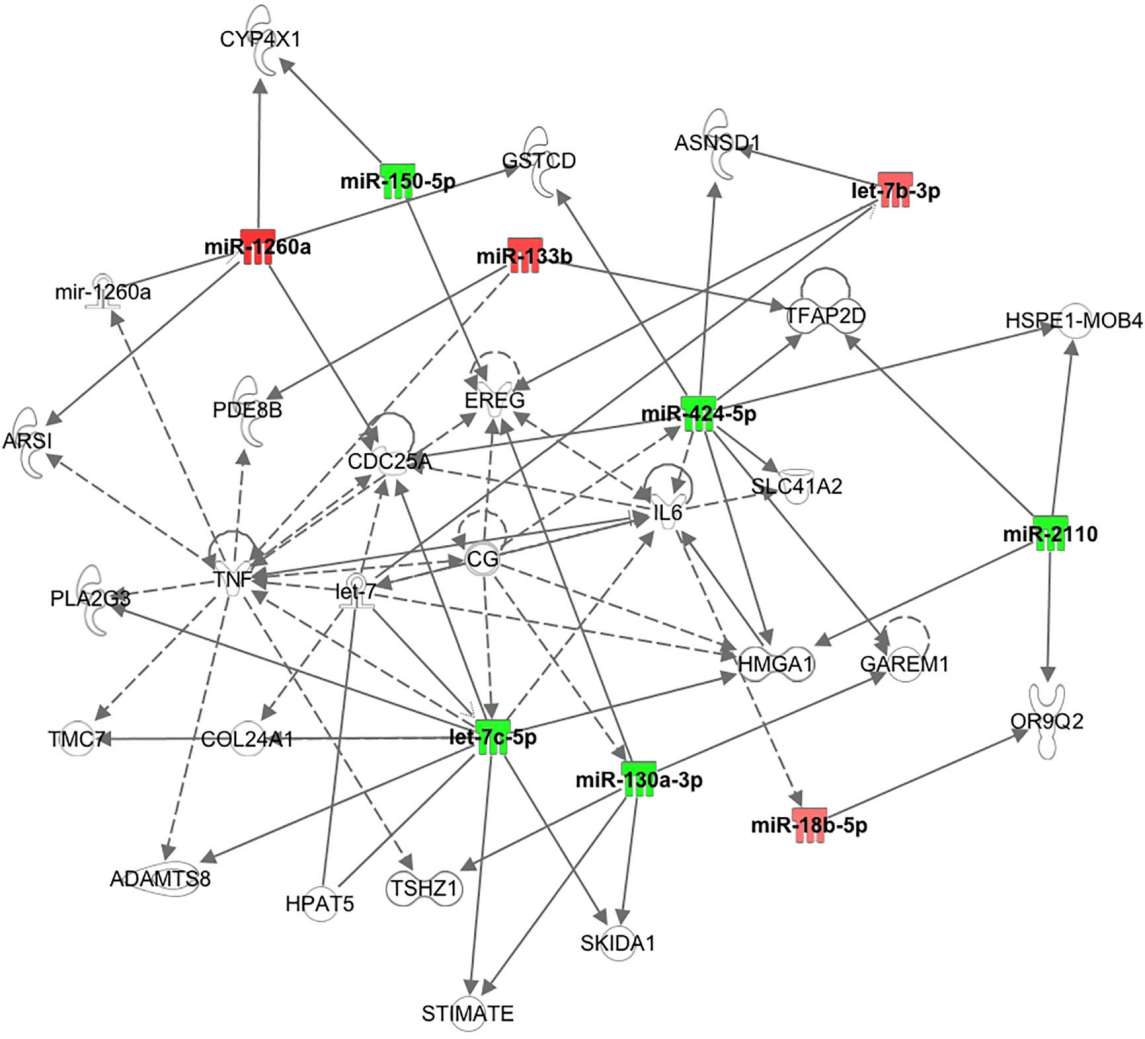
Pathway connections for the inflammatory pathways for the top 10 miRNAs showing nine were associated with the pathway relating to tumor necrosis factor and interleukin 6.

It has been shown recently that differing noncoding RNA can act in a coordinated manner to form a regulatory noncoding RNA network interacting with other noncoding RNAs, such as microRNA and circularRNA that may then effect multiple downstream biological processes (30). Therefore, Ingenuity Pathway Analysis was undertaken for the top 10 miRNAs determined here. As shown in **Figure 2**, nine of the top 10 miRNAs showed functional connectivity as part of the regulatory network of genes involved in the pathway associated with inflammation and these included miR-1260a, miR-18b-5p, miR-424-5p, let-7b-3p, miR-2110, let-7c-5p, miR-130a-3p, miR-150-5p, and miR-133b; however, CRP as a marker of inflammation did not differ between groups (**Table 1**).

## Discussion

In this non-obese, non-insulin resistant cohort of women with PCOS, four miRNAs showed significantly altered expression compared to the control women: miR-1260a, miR-18b-5p, miR-424-5p, and let-7b-3p. When obesity and insulin resistance are taken into account, it is likely that these four miRNA are specifically related to PCOS. Further, it is of interest that let-7b-3p correlated with AMH in PCOS women, as AMH has been suggested as a biomarker of PCOS though of low sensitivity (31, 32). When weight matched obese PCOS and control women are compared, the number of miRNA that differ between groups are much fewer than those previously reported in the literature (33), and none of the miRNA reported for that obese cohort differed in this study. As expected, several of the miRNA found in the obese study previously reported were associated with insulin resistance (34) whilst the four miRNA described here were not.

The strength of this study was the homogenous control and PCOS populations studied; however, this was a selected PCOS population that may not be generalizable. In addition, this pilot study was limited by having a small number of subjects, yet clearly showed that miRNAs do differ between PCOS and control women when age, BMI and insulin resistance are accounted for. However, functional studies are required to determine the role of each of the miRNA reported here.

In this study, analysis of the combined PCOS and control women revealed that miR-1260a correlated with FAI and AMH, but this correlation was not found in obesity with insulin resistance (33). It is reported that miR-1260a is associated with type 2 diabetes (35) as is miR-424 (36), whilst miR-let 7 has been associated with a reduction in insulin signaling (36). MiR-let-7b-3p correlated with BMI for these normal weight subjects, a correlation that appeared to be lost in obese subjects (33).

Recent evidence indicates that long-noncoding RNA, circular RNA and microRNA may act together as a functional regulatory network (30) and therefore the function of an individual miRNA may not be evident. In addition, with so few studies to date, individual microRNA may not obviously map its function to the disease process under study. This may be seen here for the miRNAs that differed between the women with and without PCOS: miR-1260a has been reported to be associated with prostate and lung cancer and aortic aneurysms (37–39); similarly, miR-18b-5p has been associated with oncogenic activity (40). MiR-424-5p may stimulate an immune effect through the mTOR pathway (41) and it has been shown that mTOR signaling was responsible for excessive follicle activation and growth in an animal model of PCOS (42). Let-7b-3p has been associated with several malignancies including melanoma, lung cancer and ovarian serous carcinoma (genecards.org).

The Ingenuity Pathway Analysis revealed that nine of the top 10 miRNA identified here showed functional connectivity as part of a regulatory network of genes predominantly associated with central regulators of inflammation through tumour necrosis factor (TNF) and interleukin 6 (IL6), though serum levels of both have been reported not to differ between PCOS and control women (43). Of note, CRP as a marker of inflammation did not differ between groups in this study. It has been suggested that chronic low grade inflammation may mediate the effect of sympathetic dysfunction on hyperandrogenism and insulin resistance (44).

Our findings are discrepant to others where serum miR-21 (45) and miR-6767-5p (46) are elevated, whilst miR-320 is lowered in PCOS (47); the likely explanation for this is that these miR are associated with an increased BMI (48). As noted above, miR-93 and miR-223 may play a role in insulin resistance (19) and did not differ in this study, likely because insulin resistance did not differ between the PCOS and control women.

This study indirectly supports the concept that PCOS women are characterized by different phenotypes: one hyperandrogenic (women included in this study) and the other hyperinsulinemic with insulin resistance (excluded in this study) (49). Therefore, the miRNA expression may significantly differ between these two phenotypes corroborating that the pathogenetic pathway could be different and therefore subjects may respond differently to treatment (50–52).

These results may be generalisable to a Caucasian population of non-insulin resistant women with PCOS, but clarification is needed to determine if there are ethnic differences as seen for other metabolic parameters (53).

In conclusion, only four of the 177 miRNAs expressed differed significantly in non- insulin resistant women with and without PCOS when age and BMI were matched. Let-7b-3p was related to AMH in PCOS, and to BMI as a group, whilst miR-1260a correlated with FAI. Nine of the 10 expressed miRNAs showed functional connectivity with the inflammatory pathway involving TNF and IL6.

## Data Availability Statement

The data that support the findings of this study are available upon request from the corresponding author AB aeb91011@gmail.com.

## Ethics Statement

The studies involving human participants were reviewed and approved by The Yorkshire and The Humber NRES ethical committee, UK. The patients/participants provided their written informed consent to participate in this study.

## Author contributions

AB contributed to data analysis and wrote the manuscript. VR, MB, and SHN-S performed the miRNA measurements. VR, TC, RD, NG, and MB contributed to data analysis. TS contributed to study design and supervised sample collection. SD undertook the statistical analysis and SH performed the ingenuity pathway analysis. SA designed the studies, supervised the work, contributed to data analysis, and was involved in preparation of the manuscript. All authors contributed to the article and approved the submitted version.

## Conflict of Interest

The authors declare that the research was conducted in the absence of any commercial or financial relationships that could be construed as a potential conflict of interest.
