# Supplementary figures and images for: Increased MicroRNA Levels in Women With Polycystic Ovarian Syndrome but Without Insulin Resistance: A Pilot Prospective Study
Source: Front Endocrinol (Lausanne). 2020 Sep 30;11:571357. doi: 10.3389/fendo.2020.571357 (PMC7556216; doi:10.3389/fendo.2020.571357)

**Supplemental figure 1.** Flow chart of patient recruitment, selection and study design.

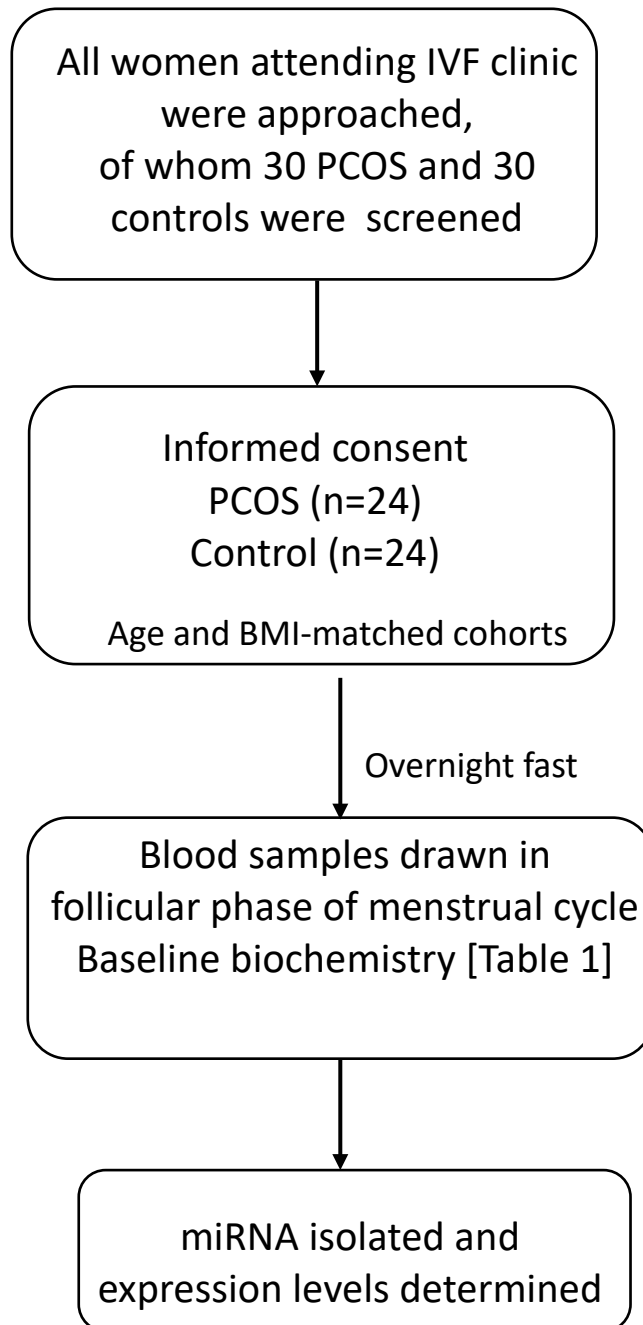

Supplement: Supplementary file 1 [file Image_1.pdf]
