# Supplementary material for: Increased MicroRNA Levels in Women With Polycystic Ovarian Syndrome but Without Insulin Resistance: A Pilot Prospective Study
Source: Front Endocrinol (Lausanne). 2020 Sep 30;11:571357. doi: 10.3389/fendo.2020.571357 (PMC7556216; doi:10.3389/fendo.2020.571357)
Supplement: Supplementary file 2 [file Table_1.docx]

Supplementary Table 1. All microRNA (176) identified and the comparison between PCOS (n=24) and control women (n=24) matched for age and body mass index. Significant differences in miR shown in bold.

Positive fold change indicates that the miRNA is upregulated in PCOS women compared to controls. Negative fold change indicates that the miRNA is downregulated in PCOS women compared to controls.

| PCOS vs Control | Fold change | FDR corrected P-Value |
| --- | --- | --- |
| **hsa-miR-1260a** | **1.94457** | **0.014676107** |
| **hsa-miR-18b-5p** | **1.32297** | **0.02868288** |
| **hsa-miR-424-5p** | **-1.40556** | **0.037762596** |
| **hsa-let-7b-3p** | **1.48463** | **0.042064683** |
| hsa-miR-2110 | -1.38854 | 0.055751654 |
| hsa-let-7c-5p | -1.37573 | 0.074952612 |
| hsa-miR-454-3p | -1.43837 | 0.075037129 |
| hsa-miR-130a-3p | 1.18537 | 0.075617532 |
| hsa-miR-150-5p | -1.38998 | 0.097941846 |
| hsa-miR-133b | 1.72953 | 0.112713908 |
| hsa-miR-362-3p | -1.14941 | 0.115295602 |
| hsa-miR-205-5p | 2.68727 | 0.11654675 |
| hsa-miR-125a-5p | -1.42585 | 0.123487845 |
| hsa-miR-22-3p | 1.09989 | 0.123654362 |
| hsa-miR-29c-3p | -1.11006 | 0.125644329 |
| hsa-miR-375 | -2.13721 | 0.12686111 |
| hsa-let-7b-5p | -1.15182 | 0.135916645 |
| hsa-miR-342-3p | -1.24089 | 0.145456549 |
| hsa-miR-136-3p | -1.56006 | 0.148088023 |
| hsa-miR-30d-5p | 1.08438 | 0.160118799 |
| hsa-miR-23a-3p | -1.18534 | 0.183629074 |
| hsa-miR-18a-5p | 1.11435 | 0.221335925 |
| hsa-miR-339-3p | 1.31377 | 0.238778263 |
| hsa-miR-185-5p | 1.11165 | 0.24666217 |
| hsa-miR-200c-3p | 1.27533 | 0.26158228 |
| hsa-miR-99b-5p | -1.29226 | 0.275732075 |
| hsa-miR-20b-5p | 1.51204 | 0.280978082 |
| hsa-miR-222-3p | -1.07225 | 0.301205601 |
| hsa-miR-874-3p | -1.07516 | 0.308301711 |
| hsa-miR-210-3p | 1.13356 | 0.310656462 |
| hsa-miR-484 | 1.0696 | 0.323237115 |
| hsa-miR-26b-5p | -1.12825 | 0.328781456 |
| hsa-miR-425-3p | -1.13873 | 0.336687644 |
| hsa-miR-132-3p | -1.22752 | 0.341992318 |
| hsa-miR-502-3p | 1.11902 | 0.346407069 |
| hsa-miR-133a-3p | 1.2313 | 0.368159053 |
| hsa-miR-328-3p | -1.12394 | 0.368271563 |
| hsa-miR-423-3p | -1.13319 | 0.374000122 |
| hsa-miR-324-5p | -1.1159 | 0.379133003 |
| hsa-miR-361-5p | -1.08445 | 0.385044829 |
| hsa-let-7d-5p | -1.15162 | 0.385321495 |
| hsa-miR-766-3p | -1.33858 | 0.386712195 |
| hsa-miR-33a-5p | -1.17689 | 0.395200848 |
| hsa-miR-29a-3p | -1.12613 | 0.396780307 |
| hsa-miR-140-5p | 1.11122 | 0.409945732 |
| hsa-miR-192-5p | 1.11718 | 0.411272903 |
| mmu-miR-378a-3p | -1.07742 | 0.414910229 |
| hsa-miR-145-5p | -1.12308 | 0.416441983 |
| hsa-miR-146b-5p | -1.22231 | 0.416474958 |
| hsa-miR-106b-3p | 1.26474 | 0.422158793 |
| hsa-miR-324-3p | 1.05296 | 0.427182787 |
| hsa-let-7a-5p | -1.08068 | 0.427656244 |
| hsa-miR-374b-5p | -1.22774 | 0.428618994 |
| hsa-miR-374a-5p | -1.17214 | 0.434749889 |
| hsa-miR-26a-5p | -1.10574 | 0.442307015 |
| hsa-miR-181a-5p | -1.11793 | 0.451129749 |
| hsa-miR-223-3p | -1.12659 | 0.453756615 |
| hsa-miR-382-5p | 1.29549 | 0.45463618 |
| hsa-miR-590-5p | 1.06065 | 0.469190046 |
| hsa-miR-15b-3p | 1.067 | 0.469496306 |
| hsa-miR-148a-3p | -1.07661 | 0.474246866 |
| hsa-miR-148b-3p | 1.03796 | 0.479417343 |
| hsa-let-7i-5p | -1.04439 | 0.485693095 |
| hsa-miR-34a-5p | 1.15959 | 0.489236765 |
| hsa-miR-7-5p | -1.17605 | 0.492687834 |
| hsa-miR-193a-5p | -1.19348 | 0.494857816 |
| hsa-miR-10b-5p | 1.19907 | 0.506651678 |
| hsa-miR-338-3p | -1.12704 | 0.506718915 |
| hsa-miR-16-5p | 1.06732 | 0.507263936 |
| hsa-let-7g-5p | 1.19355 | 0.512974586 |
| hsa-miR-19a-3p | 1.04438 | 0.518483023 |
| hsa-miR-99a-5p | 1.10971 | 0.550683665 |
| hsa-let-7e-5p | -1.21684 | 0.550801081 |
| hsa-miR-7-1-3p | -1.17873 | 0.552490112 |
| hsa-miR-151a-5p | -1.07501 | 0.555735123 |
| hsa-miR-505-3p | 1.11008 | 0.564687034 |
| hsa-miR-130b-3p | 1.12003 | 0.564766295 |
| hsa-miR-92b-3p | -1.16995 | 0.566074581 |
| hsa-miR-532-5p | 1.05625 | 0.570933307 |
| hsa-miR-106a-5p | 1.04402 | 0.571108652 |
| hsa-miR-122-5p | 1.19614 | 0.579266626 |
| hsa-miR-21-5p | -1.02828 | 0.585327999 |
| hsa-miR-140-3p | 1.04474 | 0.586871387 |
| hsa-miR-23b-3p | -1.06497 | 0.589525986 |
| hsa-miR-423-5p | -1.03428 | 0.594765766 |
| hsa-miR-501-3p | -1.13112 | 0.60185013 |
| hsa-miR-543 | 1.15416 | 0.602149265 |
| hsa-miR-125b-5p | -1.08049 | 0.612138659 |
| hsa-miR-142-5p | -1.05958 | 0.615075398 |
| hsa-miR-30e-5p | -1.03515 | 0.615563855 |
| hsa-let-7f-5p | 1.0662 | 0.615793452 |
| hsa-miR-660-5p | -1.04388 | 0.616064513 |
| hsa-miR-197-3p | -1.0773 | 0.620315182 |
| hsa-miR-335-5p | -1.09952 | 0.630122792 |
| hsa-miR-151a-3p | 1.10789 | 0.635486964 |
| hsa-miR-24-3p | -1.06036 | 0.63674976 |
| hsa-miR-363-3p | 1.04904 | 0.647955806 |
| hsa-miR-19b-3p | 1.03046 | 0.651986469 |
| hsa-miR-339-5p | 1.08046 | 0.657612819 |
| hsa-miR-29b-3p | 1.03785 | 0.658698028 |
| hsa-miR-128-3p | 1.04141 | 0.661032197 |
| hsa-miR-495-3p | -1.14706 | 0.666937619 |
| hsa-miR-22-5p | 1.04725 | 0.668050381 |
| hsa-miR-320c | 1.05232 | 0.671552368 |
| hsa-miR-127-3p | -1.17425 | 0.67292507 |
| hsa-miR-194-5p | 1.07828 | 0.67501072 |
| hsa-miR-320a | 1.02939 | 0.679386212 |
| hsa-miR-101-3p | 1.03123 | 0.684024849 |
| hsa-miR-584-5p | -1.07613 | 0.69426845 |
| hsa-miR-200a-3p | -1.17293 | 0.697466849 |
| hsa-miR-885-5p | 1.20607 | 0.69902414 |
| hsa-miR-409-3p | -1.07272 | 0.69955026 |
| hsa-miR-100-5p | 1.14083 | 0.700525976 |
| hsa-miR-15b-5p | -1.07008 | 0.711462788 |
| hsa-miR-376a-3p | 1.1242 | 0.71461313 |
| hsa-miR-146a-5p | 1.05909 | 0.718126273 |
| hsa-miR-421 | -1.08432 | 0.727100367 |
| hsa-let-7d-3p | -1.03506 | 0.727628704 |
| hsa-miR-92a-3p | 1.03234 | 0.727882587 |
| hsa-miR-126-5p | 1.02623 | 0.738245071 |
| hsa-miR-30a-5p | -1.09757 | 0.740658071 |
| hsa-miR-326 | -1.07797 | 0.741331351 |
| hsa-miR-215-5p | 1.04244 | 0.746651158 |
| hsa-miR-30e-3p | 1.09528 | 0.747167373 |
| hsa-miR-28-5p | -1.0934 | 0.74931779 |
| hsa-miR-106b-5p | 1.0282 | 0.753575742 |
| hsa-miR-142-3p | 1.04541 | 0.760087783 |
| hsa-miR-320b | 1.02195 | 0.761285833 |
| hsa-miR-486-5p | 1.03697 | 0.761756205 |
| hsa-miR-191-5p | -1.02461 | 0.765215973 |
| hsa-miR-877-5p | -1.06419 | 0.76557156 |
| hsa-miR-195-5p | 1.07941 | 0.773619724 |
| hsa-miR-136-5p | -1.08114 | 0.775700422 |
| hsa-miR-223-5p | 1.07062 | 0.783574492 |
| hsa-miR-451a | 1.03146 | 0.783886149 |
| hsa-miR-574-3p | 1.0592 | 0.796095048 |
| hsa-miR-141-3p | 1.06746 | 0.798740721 |
| hsa-miR-126-3p | 1.01847 | 0.800683011 |
| hsa-miR-25-3p | 1.02425 | 0.805277281 |
| hsa-miR-199a-3p | 1.04004 | 0.822139818 |
| hsa-miR-155-5p | 1.04611 | 0.82839705 |
| hsa-miR-652-3p | -1.01799 | 0.832769749 |
| hsa-miR-154-5p | -1.07128 | 0.832975521 |
| hsa-miR-28-3p | 1.05632 | 0.83622555 |
| hsa-miR-629-5p | -1.03881 | 0.845071443 |
| hsa-miR-425-5p | 1.01576 | 0.846291993 |
| hsa-miR-103a-3p | -1.01746 | 0.847874299 |
| hsa-miR-144-3p | -1.02612 | 0.855964302 |
| hsa-miR-15a-5p | 1.01369 | 0.860035148 |
| hsa-miR-17-5p | -1.0277 | 0.870408817 |
| hsa-miR-32-5p | 1.01162 | 0.88270328 |
| hsa-miR-221-3p | -1.02586 | 0.889528723 |
| hsa-miR-30c-5p | 1.01462 | 0.894035327 |
| hsa-miR-376c-3p | 1.01941 | 0.894856968 |
| hsa-miR-186-5p | 1.01585 | 0.897273927 |
| hsa-miR-27a-3p | -1.018 | 0.902091831 |
| hsa-miR-93-3p | 1.01659 | 0.907902825 |
| hsa-miR-365a-3p | 1.03264 | 0.910838748 |
| hsa-miR-93-5p | 1.00833 | 0.914709171 |
| hsa-miR-1 | 1.02742 | 0.91652721 |
| hsa-miR-107 | -1.00814 | 0.920575127 |
| hsa-miR-144-5p | -1.02535 | 0.922845893 |
| hsa-miR-152-3p | 1.01017 | 0.931776309 |
| hsa-miR-139-5p | -1.02292 | 0.932518733 |
| hsa-miR-27b-3p | -1.00966 | 0.943914078 |
| hsa-miR-335-3p | 1.02079 | 0.945032932 |
| hsa-miR-30b-5p | 1.0061 | 0.947031193 |
| hsa-miR-16-2-3p | 1.00651 | 0.958848285 |
| hsa-miR-320d | 1.00389 | 0.96906848 |
| hsa-miR-301a-3p | 1.00538 | 0.970236146 |
| hsa-miR-143-3p | 1.00639 | 0.970749061 |
| hsa-miR-485-3p | 1.00417 | 0.982935497 |
| hsa-miR-532-3p | -1.00182 | 0.987918768 |
| hsa-miR-199a-5p | 1.00348 | 0.989815009 |
| hsa-miR-331-3p | 1.00126 | > 0.99 |
| hsa-miR-497-5p | 1.00213 | > 0.99 |
| hsa-miR-20a-5p | 1.00055 | > 0.99 |
